# Caregiving of Older Adults: Challenges and Opportunities

**DOI:** 10.1093/geroni/igaf122.795

**Published:** 2025-12-31

## Abstract

Presenters address caregiver burden challenges and strategies to improve the caregiving experience.

